# Supplementary material for: From sequence to enzyme mechanism using multi-label machine learning
Source: BMC Bioinformatics. 2014 May 19;15:150. doi: 10.1186/1471-2105-15-150 (PMC4229970; doi:10.1186/1471-2105-15-150)
Supplement: Additional file 2 — Java code of ml2db. Additional file ml2db_code.tar.gz contains the Java source code to run the multi-label machine learning experiments and save the results to database. The code’s Javadoc is included. [file 1471-2105-15-150-S2.zip › additional file 2/ml2db/ml2db/doc/main/ml2db/al/utility/neighbours/Neighbourhood.html]

Neighbourhood


JavaScript is disabled on your browser.


- Overview
- Package
- Class
- Use
- Tree
- Deprecated
- Index
- Help

- Prev Class
- Next Class

- Frames
- No Frames

- All Classes

- Summary:
- Nested |
- Field |
- Constr |
- Method

- Detail:
- Field |
- Constr |
- Method


main.ml2db.al.utility.neighbours

## Class Neighbourhood

- java.lang.Object
- - weka.core.AbstractInstance
  - - weka.core.SparseInstance
    - - main.ml2db.mulan.mulanarff.IdInstance
      - - main.ml2db.al.utility.neighbours.Neighbourhood

- All Implemented Interfaces:
  :   java.io.Serializable, java.lang.Comparable<IdInstance>, weka.core.Copyable, weka.core.Instance, weka.core.RevisionHandler

  ---

    

  ```
  public class Neighbourhood
  extends IdInstance
  ```

  Contains information about: an instance, how much it is needed in this data
  set (the instances it is the nearest neighbour of), and how much it is
  labelled (whether it or its nearest or further neighbours are already
  labelled or partially known). For now, only tested for number of nearest
  neighbours k=1.

  Version:
  :   21 Jul 2011

  Author:
  :   Luna De Ferrari ldf(at)st-andrews.ac.uk

  See Also:
  :   Serialized Form

- - ### Constructor Summary

    Constructors

    Constructor and Description

    `Neighbourhood(java.lang.String identifier,
    weka.core.Instance instance)`
  - ### Method Summary

    Methods

    | Modifier and Type | Method and Description |
    | --- | --- |
    | `void` | `addNeighbour(Neighbourhood neighbour)` |
    | `double` | `getInformationPerNeighbour()` Returns the fraction of information that each neighbour contributes to this instance (1 / number of neighbours ) |
    | `java.util.Vector<Neighbourhood>` | `getIsNeededBy()` |
    | `java.util.Vector<Neighbourhood>` | `getNeededNeighbours()` |
    | `int` | `getNumberOfNearestNeighbours()` |
    | `int` | `getNumberOfNeedy()` |
    | `int` | `getNumberOfUnlabelledNeedy()` Only consider instances that still need a label (exclude labelled instances). |
    | `double` | `getPercentPotentiallyLabelled()` |
    | `boolean` | `isLabelled()` |
    | `void` | `setIsNeededBy(Neighbourhood instance)` |
    | `void` | `setLabelled(boolean labelled)` |
    | `void` | `setNumberOfNearestNeighbours(int numberOfNearestNeighbours)` |
    | `void` | `setPercentPotentiallyLabelled(double percentPotentiallyLabelled)` |

    - ### Methods inherited from class main.ml2db.mulan.mulanarff.IdInstance

      `compareTo, equals, equals, generateAllAttributeNamesString, generateNonZeroAttributesNames, getString, getWekaInstance, identifier, setWekaInstance, toIdString, toSparseAttributesString, toString`
    - ### Methods inherited from class weka.core.SparseInstance

      `copy, getRevision, index, locateIndex, main, mergeInstance, numAttributes, numValues, replaceMissingValues, setValue, setValueSparse, toDoubleArray, toStringNoWeight, value`
    - ### Methods inherited from class weka.core.AbstractInstance

      `attribute, attributeSparse, classAttribute, classIndex, classIsMissing, classValue, dataset, deleteAttributeAt, enumerateAttributes, equalHeaders, equalHeadersMsg, hasMissingValue, insertAttributeAt, isMissing, isMissing, isMissingSparse, numClasses, relationalValue, relationalValue, setClassMissing, setClassValue, setClassValue, setDataset, setMissing, setMissing, setValue, setValue, setValue, setWeight, stringValue, stringValue, toString, toString, value, valueSparse, weight`
    - ### Methods inherited from class java.lang.Object

      `equals, getClass, hashCode, notify, notifyAll, wait, wait, wait`

- - ### Constructor Detail


    - #### Neighbourhood

      ```
      public Neighbourhood(java.lang.String identifier,
                   weka.core.Instance instance)
      ```
  - ### Method Detail


    - #### addNeighbour

      ```
      public void addNeighbour(Neighbourhood neighbour)
      ```


    - #### getInformationPerNeighbour

      ```
      public double getInformationPerNeighbour()
      ```

      Returns the fraction of information that each neighbour contributes to
      this instance (1 / number of neighbours )

      Returns:
      :   the fraction of information that each neighbour contributes to
          this instance


    - #### getIsNeededBy

      ```
      public java.util.Vector<Neighbourhood> getIsNeededBy()
      ```


    - #### getNeededNeighbours

      ```
      public java.util.Vector<Neighbourhood> getNeededNeighbours()
      ```


    - #### getNumberOfNearestNeighbours

      ```
      public int getNumberOfNearestNeighbours()
      ```


    - #### getNumberOfNeedy

      ```
      public int getNumberOfNeedy()
      ```


    - #### getNumberOfUnlabelledNeedy

      ```
      public int getNumberOfUnlabelledNeedy()
      ```

      Only consider instances that still need a label (exclude labelled
      instances).

      Returns:


    - #### getPercentPotentiallyLabelled

      ```
      public double getPercentPotentiallyLabelled()
      ```


    - #### isLabelled

      ```
      public boolean isLabelled()
      ```


    - #### setIsNeededBy

      ```
      public void setIsNeededBy(Neighbourhood instance)
      ```


    - #### setLabelled

      ```
      public void setLabelled(boolean labelled)
      ```


    - #### setNumberOfNearestNeighbours

      ```
      public void setNumberOfNearestNeighbours(int numberOfNearestNeighbours)
      ```


    - #### setPercentPotentiallyLabelled

      ```
      public void setPercentPotentiallyLabelled(double percentPotentiallyLabelled)
      ```


- Overview
- Package
- Class
- Use
- Tree
- Deprecated
- Index
- Help

- Prev Class
- Next Class

- Frames
- No Frames

- All Classes

- Summary:
- Nested |
- Field |
- Constr |
- Method

- Detail:
- Field |
- Constr |
- Method
